# Current Diabetes Technology: Striving for the Artificial Pancreas

**DOI:** 10.3390/diagnostics9010031

**Published:** 2019-03-15

**Authors:** Natalie Allen, Anshu Gupta

**Affiliations:** Division of Pediatric Endocrinology, Virginia Commonwealth University, Children’s Hospital of Richmond, Richmond, VA 23298, USA; anshu.gupta@vcuhealth.org

**Keywords:** type 1 diabetes, continuous glucose monitor, insulin pump, continuous insulin infusion system, hybrid closed-loop system, diabetes technology

## Abstract

Diabetes technology has continually evolved over the years to improve quality of life and ease of care for affected patients. Frequent blood glucose (BG) checks and multiple daily insulin injections have become standard of care in Type 1 diabetes (T1DM) management. Continuous glucose monitors (CGM) allow patients to observe and discern trends in their glycemic control. These devices improve quality of life for parents and caregivers with preset alerts for hypoglycemia. Insulin pumps have continued to improve and innovate since their emergence into the market. Hybrid closed-loop systems have harnessed the data gathered with CGM use to aid in basal insulin dosing and hypoglycemia prevention. As technology continues to progress, patients will likely have to enter less and less information into their pump system manually. In the future, we will likely see a system that requires no manual patient input and allows users to eat throughout the day without counting carbohydrates or entering in any blood sugars. As technology continues to advance, endocrinologists and diabetes providers need to stay current to better guide their patients in optimal use of emerging management tools.

## 1. Introduction

Type 1 diabetes (T1DM) is an autoimmune disease in which the β cells of the pancreas are damaged and subsequently destroyed. Since these cells are critical in the production of insulin hormone, this leads to a state of insulin deficiency. Landmark studies, such as the Diabetes Control and Complications Trial (DCCT) and the ongoing Epidemiology of Diabetes Interventions and Complications (EDIC) follow up study, have conclusively proven the long-term benefits of early and intensive blood glucose control on the future development of diabetes-related complications, such as heart, eye, kidney, and nerve disease, and improved life expectancy [1]. As a result, frequent blood glucose (BG) checks and multiple daily insulin injections have become standard of care in T1DM management. These advances have brought forth challenges of managing a chronic illness as well as opportunities to apply technological advances from engineering to medicine.

Diabetes was referenced as early as 1500 B.C. in Egyptian manuscripts as a disease noted for increased urination. The techniques for diagnosis of T1DM were described by the middle of the 16th century when it was recognized by detection of sugar in the urine [2]. However, insulin was not discovered until 1921, when Charles Best and Frederick Banting used canine pancreas extracts to successfully treat diabetic dogs. In 1923, Eli Lilly introduced the first insulin product, a further purified pancreas extract, to the market [3]. Since that time, many different insulins and innovative insulin delivery devices have been developed from insulin pens to insulin pumps. Both diagnostic tools and treatments have continued to evolve in the 20th and 21st centuries.

The field of glucose monitoring and insulin administration technology has transformed in the past two decades. Patients in the developed world use palm-size or smaller handheld glucometers as a standard of care for managing their diabetes. Additionally, continuous glucose monitor (CGM) devices, which allow patients to wear a sensor and obtain frequent automated BG readings, are quickly gaining a central place in T1DM management. Furthermore, integrated insulin pump and CGM systems are now increasingly available; this has improved the ease of insulin delivery in addition to prevention of severe hypoglycemia, one of the most feared complications of treatment with insulin. As the technology in diabetes care continues to evolve, providers must be up-to-date on available systems and advocate for the care of their patients. In this review article, we discuss currently available and shortly anticipated diabetes technology available for T1DM patients within the United States (US) and Europe with the goal of providing a historical perspective and an update to clinicians caring for patients with diabetes so they can facilitate discussions on therapeutic options with their patients.

## 2. Methods

A thorough review of the existing literature was conducted via PubMed and Google Scholar. Keywords included were ‘diabetes’, ‘T1DM’, ‘continuous glucose monitor’, ‘CGM’, ‘insulin pump’, ‘continuous insulin infusion system’, ‘hybrid closed loop system’, ‘bihormonal’, ‘Abbott Freestyle Libre’, ‘Dexcom’, ‘Medtronic’, ‘Tandem’, ‘OmniPod’, ‘iLet’. Relevant studies from 2007–2019 published in the English language were reviewed and included.

## 3. Continuous Glucose Monitors

Continuous glucose monitors are devices that help patients with diabetes to monitor their glucose levels over time. They allow for dynamic information that indicates direction of glucose change allowing the user to observe the influence of diet, exercise, and insulin dosing on the patient’s glucose level in real-time. Additionally, they can be used in conjunction with insulin pumps to better guide insulin therapy. Studies have shown that CGM use can increase time spent in normoglycemia and decrease the frequency and severity of hypoglycemia episodes [4,5]. In a randomized study of adults with hypoglycemia unawareness, researchers found that patients in the CGM group had a 72% reduction in hypoglycemia events [6]. Alarms may be set on some of these devices to alert the user to urgent low glucose levels or elevated glucose levels. Many devices even allow sharing of data to multiple smartphones providing parents with access to remotely monitor their child’s glucose levels and allowing physicians to view their patient’s glucose trends and accordingly adjust insulin therapy [7].

Medtronic Minimed CGM was the first CGM system approved by the FDA in 1999 after it had been tested in Caucasian adults with T1DM [8]. This first available system was not meant to be used in real-time and BG trends could only be evaluated after the window of therapy [9]. Additionally, the FDA specifically stipulated that this device be used in conjunction with regular finger-stick BG checks. Over the 20 years since the introduction of the first CGM system to market, many new systems and updates have been introduced and have revolutionized management of T1DM. In early 2017, the Centers for Medicare & Medicaid Services (CMS) determined that CGM systems that are FDA approved for treatment decisions could be eligible for Medicare coverage [10]. Recently the American Diabetes Association has endorsed use of CGMs in children and adolescents with T1DM to help improve glucose control while minimizing risk of hypoglycemia [11].

A CGM consists of an electrode sensor placed subcutaneously, a transmitter, and a receiver. The sensor detects glucose levels by electrochemical detection of glucose [9]. This sensor measures the glucose levels in the interstitial fluid and is attached to a transmitter that sends the data to a receiver. The glycemic data and trends are displayed on the receiver or on the pump screen of part of an integrated system. 

Various sensors that have been used include biosensors, which involve the use of natural oxygen cofactor, artificial redox mediators, direct electron transfer between glucose oxidase and the electrode, and direct electro-oxidation of glucose [12]. The most commonly used method in CGM technology today uses electrochemical sensors because of increased sensitivity and accuracy [13]. Typically, this will use glucose oxidase (GOx) to detect glucose levels. This particular enzyme electrochemical sensor reaction has gained widespread use because GOx remains stable at variable pH values and temperatures. GOx, with the aid of a cofactor, facilitates transfer of electrons and production of hydrogen peroxide. The electrochemical sensor detects the number of electron transfers and, therefore, the number of glucose molecules present. Oxygen is important for this reaction to occur in the subcutaneous tissue. In the setting of poor perfusion or hypoxia, sensors may not be able to accurately detect glucose levels without appropriate levels of oxygen present [13,14]. Fluorescence-based sensors have also been used and are discussed in further detail below.

Current recommendations for BG monitoring advise 6–10 BG readings daily to guide insulin management of T1DM patients [15]. CGM systems allow for readings every five minutes approximately throughout the day and night and, thus, provide information about accurate trends that previously were not accessible. Additionally, with some of the new systems, routine BG checks for mealtime insulin dosing may be replaced with CGM readings. At this point in time, there is a recommendation to check a finger-stick BG manually to confirm hypoglycemia or if clinically warranted; however, this may change in the future as sensor accuracy continues to improve. 

Accuracy of systems is a critical aspect of CGM use and system calibrations have typically been required to ensure accuracy of readings. With typical home glucometer use, there is approximately a 20% margin of error for BG measurements [16]. Additionally, it is important to remember, that finger-stick BG values measure glucose from capillary blood while CGM sensors measure glucose from the interstitial fluid in the subcutaneous fat. These compartments do have slightly different glucose values especially during periods of rapid change in glycemia. This discrepancy in measurement values is known as ‘lag time’ in which the interstitial fluid glucose levels are lagging behind the blood values by a median of 6.8 min and up to almost 10 min in some cases [17,18]. The accuracy of systems is typically reported as a mean absolute relative difference (MARD) which is determined using data from clinical trials and calculated by using the difference in the CGM measurement and concurrent reference measurement of glucose used in the study [19]. Currently available CGM systems are discussed further in the sections below with system comparisons outlined in Table 1.

### 3.1. Standalone

#### 3.1.1. Abbott Freestyle CGM Systems

Abbott’s first CGM was the Freestyle Navigator and was initially FDA approved in 2008. This product was then subsequently pulled when Abbott’s newer system the Freestyle Libre Flash CGM, which did not require patient calibration with glucose entry, came out in 2014. Recently, the system has been updated and may be worn for 14 days prior to requiring a new sensor. The sensors are factory calibrated and require no calibration for the duration of the 14-day wear period [14]. There is a 1 h warm up period for the device to start generating data. The data are transferred to the reader when it is brought in proximity to the sensor. The reader then displays the current blood sugar with an arrow indicating the direction of the glucose trend and the readings over the last 8 h. The sensor is able to hold 8 h of data and data may be lost if the reader is not used in more than 8 h. The reader can hold data for up to 90 days and may be uploaded using device software or accessed via an approved smartphone device and the Freestyle LibreLink application. The Freestyle Libre has been approved for insertion in the arm [24]. While in the US, it is approved by the FDA in patients greater than 18 years old, it has been cleared for pediatric patients ages four years and up in Europe and Australia [25].

#### 3.1.2. Dexcom CGM Systems

Dexcom is a company that focuses solely on CGM technology for diabetes patients. They first came out with their Short-Term Sensor (STS) system in 2006. In 2015, they released the Dexcom G4 Platinum CGM system. This system consists of a small sensor that measures glucose levels subcutaneously, a transmitter that is attached to the top of the sensor, and a receiver which displays the glucose readings and trends. With the introduction of their G4 system, Dexcom added share technology so that BG readings and trends may be monitored remotely via smartphone. The system originally worked exclusively with iOS systems, but is now compatible with some android devices. The Dexcom G5 system allowed users to bypass the receiver altogether and directly send data from the sensor/transmitter unit to their smartphone. The newest iteration, the Dexcom G6, can integrate with Tandem and display CGM data on the Tandem X2 insulin pump.

In March 2018, Dexcom released the G6 CGM system that is FDA approved for patients as young as two years old [26]. The G6 is factory calibrated with no need for calibration or finger-sticks to make clinical decisions based on FDA approval. Additionally, the G6 system is approved for wear up to 10 days. Through the smartphone application or receiver, alarms can be set for glucose target ranges to alert the user to episodes of hypoglycemia or hyperglycemia. Additionally, data may be shared with more than one smartphone device allowing parents and caregivers to remotely monitor the patient’s glucose measurements.

#### 3.1.3. Senseonics Eversense CGM System

Senseonics Eversense is the only implantable CGM device currently on the market. The device consists of an implantable sensor, removable transmitter, and a smartphone application. The transmitter activates the sensor which flashes an LED light source exciting the fluorescent polymer coating on the outside of the sensor. Glucose in the interstitial fluid will reversibly bind to the coating and alter the amount of light emitted by the polymer coating. The amount of emitted light is proportional to interstitial glucose concentration and is measured by photodetectors inside the sensor. The smart transmitter is attached to the skin with an adhesive, sitting directly over the sensor, and vibrates for alerts including hypoglycemia even when the smartphone is not in range. The transmitter is rechargeable and does not need to be replaced every three months with the implantable sensor. The implantation process can be done in the outpatient office setting by trained health care professionals. There is a 24-h warm up period after the sensor is initially placed followed by an initialization period that requires multiple calibrations within another 12-h period. Thereafter, the system requires two calibrations daily and advises finger-stick checks for treatment decisions regarding hypoglycemia or hyperglycemia. The transmitter does require charging daily that takes approximately 15 min to complete. The transmitter is water resistant up to a depth of 3.3 feet for 30 min at a time; however, the user needs to avoid submersion for the first five days of wear [27].

In the PRECISE study, Eversense was tested in 71 adults with type 1 and type 2 diabetes in multiple European countries. The mean absolute relative difference (MARD), a standard measure of CGM accuracy, was 11.1% with a similar error profile to current CGM technology [28]. Following the initial study, the calibration methods were updated and a follow up study called PRECISE II was performed. This study was a multicenter US, nonrandomized, blinded trial in 90 adults and reported a MARD of 8.8% [23]. The in-development closed loop system iLet works in conjunction with the Eversense CGM system [29].

### 3.2. CGM Integrated with Insulin Pumps

#### Medtronic CGM Systems

As mentioned before, the Medtronic Minimed CGM was released in 1999 and was the first FDA-approved CGM device available. This system included a subcutaneous glucose sensor, a monitor, a connecting cable, and a ComStation compatible with Windows 95. It was updated to include alarms for hyperglycemia and hypoglycemia with a new device name, the Guardian Real-time CGM system. The next Medtronic system approved in 2016 was the Medtronic Minimed iPro2 system and was designed to be compatible with the Medtronic 530G insulin pump system. 

The most recent Medtronic CGM system is the Guardian Sensor 3 system and works in conjunction with the Medtronic 670G insulin pump utilizing hybrid closed loop technology or with the Medtronic 630G insulin pump providing low glucose suspend. The Guardian Sensor 3 requires calibration at least twice daily, but recommends 3–4 calibrations per day for optimal accuracy. The new system does not require the purchase of a separate receiver, but directly transmits data via Bluetooth to the Medtronic insulin pump and/or an approved smartphone with the Medtronic application. The Medtronic Guardian Sensor 3 CGM can also be used in conjunction with insulin injections and features prediction of highs and lows with an alert 10–60 min prior to hyper- or hypoglycemia [30]. This sensor can alert patients to glucose changes via their smartphone or smartwatch similar to SMS messaging.

### 3.3. Limitations

Continuous glucose monitor systems have improved the monitoring of BGs for T1DM patients and allowed medical providers, caregivers, and patients access to BG trends that would otherwise be missed. However, there are significant limitations that must be considered with CGM systems. The cost of a CGM system can be prohibitive for patients who are uninsured or for whom insurance does not cover CGM. There are not only initial costs for the transmitter and receiver set up, but also monthly costs for sensor and transmitter replacement. Typically, sensors will last from 7–14 days depending on the CGM model. Transmitters additionally may need to be replaced monthly to a couple times per year. Since 2017, Medicaid has started to cover CGM costs with commercial insurance groups beginning to cover CGM prior to that time. 

Continuous glucose monitors also require an additional body site for insertion away from an insulin pump site or injection site. In children and adults, CGM devices only have approval in particular sites. For the pediatric population, typically this is in the abdomen or gluteus. Patients however often need to interchange between multiple insertion sites most often in the abdomen, gluteus, or bilateral arms. There seems to be no difference in accuracy between sites among pediatric patients [31]. CGM systems over time have shown a variable degree of water resistance; therefore, patients need to pay attention to specific system capabilities: Abbott Freestyle Libre is water resistant to 3 feet for up to 30 min, Dexcom G6 sensor/transmitter are water resistant, Medtronic Guardian Sensor 3 is water resistant to 8 feet for up to 30 min, and Senseonics Eversense is water resistant to 3.3 feet for up to 30 min [25,26,27,30]. Due to the need for Bluetooth technology to transmit data to the receiver, CGMs may lose contact during water-based activities, such as showers, baths, and swimming [26]. During times when data is not being received, alerts based on glucose values will not be functional.

While glucose measurements are comparable during times of homeostasis between CGM systems and finger-stick glucose values, CGMs can lag behind when rapid glucose changes occur. Like all populations, patients with T1DM are encouraged to engage in physical activity for their overall health. However, sudden changes in glucose might pose a challenge to current CGM capacity to accurately depict glucose trends and forewarn patients of impending hypoglycemia during or after physical activity. Zaharieva et al. reported a case study where they had a 40-year-old patient with T1DM who wore a Medtronic 670G pump and CGM integrated system, a Dexcom G5, and an Abbott Freestyle Libre during exercise. The patient performed one hour of moderate intensity running and monitored glucose values through the CGM systems and finger-stick measurements. All three of the CGM systems lagged behind finger-stick values when glucose values dropped during exercise with the largest difference in glucose levels noted in the first 30 min of exercise [32]. Additional studies have supported the finding that discrepancies between CGM and finger-stick values are greatest during exercise and return to previous accuracy within a couple hours after aerobic exercise [33].

### 3.4. Future Directions

Continuous glucose monitors have improved immensely in the last 20 years of clinical care. Future CGMs will need to improve upon the lag time of current devices to allow for better monitoring and management during physical exercise and periods of rapid glucose changes. As these products continue to develop, sensors and transmitters will likely become smaller and lower profile to skin for ease of wearability. Additionally, new adhesive strategies need to be employed to keep these devices in place as the lifetime of a sensor continues to improve. CGMs are already being used in hybrid closed-loop systems with insulin pumps; simultaneously, more collaborations are ongoing to make currently available CGM devices compatible with other independent insulin pumps with the goal of developing closed loop and suspend before low systems in the near future.

## 4. Insulin Pumps

Currently, it is estimated that more than one million people worldwide use an insulin pump for diabetes management including nearly 400,000 patients with T1DM in the US [34]. Insulin pumps are small devices that deliver short acting insulin subcutaneously via a small cannula self-placed by the patient every few days. They are designed to provide a near-continuous low dose of insulin delivered frequently in small boluses to mimic the actions of the β cells of the pancreas and also allow for bolus delivery of insulin at meal times or when needed for rapid correction of glucose. Insulin is given through a small tube called a cannula that is inserted into the subcutaneous tissue with a small needle and taped to the skin. Some insulin pump systems have tubing that runs from the device’s insulin reservoir to the insertion site and some insulin pumps are tubing free. Tubing free systems stick directly to the skin with a cannula inserted into the subcutaneous tissue below the pump.

The pump itself is a battery-powered programmable device that holds multiple settings. Providers and patients are able to program multiple insulin delivery settings based on the time of day and in multiple profiles. Within a profile, there will be basal rates for 24-h, and typically a programmed carb ratio and insulin sensitivity factor with a target glucose. Additionally, settings may include insulin action time to allow the pump to calculate current insulin activity when giving additional boluses. Most pumps contain an insulin reservoir that typically is in a cylindrical shape. When the user programs the pump to deliver insulin, the plunger is pushed by the pump’s internal mechanisms to precisely deliver the amount of insulin desired. The Tandem insulin pumps, however, use a bag reservoir for the insulin to allow for a smaller pump size.

The first insulin pump was designed by Dr. Arnold Kadish in the 1960s and was the size of a large backpack. In the 1970s, Dean Kamen introduced the AS2C, also known as the ‘blue brick’, and later the ‘autosyringe.’ These initial systems were large and often difficult to use even requiring a screwdriver to adjust insulin doses. By the mid-1980s, smaller and more precise systems were starting to emerge [35]. A large producer of insulin pumps, Animas announced in 2017 that it would be discontinuing insulin pump production and support. The insulin pump market has continued to evolve and now has three major companies producing insulin pumps in the US: Medtronic, Insulet, and Tandem. There are multiple other insulin pump brands out on the market and in development worldwide, including Cellnovo, Kaleido, Roche Diagnostics Accu-Chek, and Sooil Dana Diabecare systems discussed below.

A major advantage of insulin pumps is that they allow patients to avoid multiple daily injections. Some pumps have even featured remote bolus programming allowing for parents of pediatric patients a convenient way to dose insulin for active kids. Additionally, insulin pumps allow for variable basal rates throughout the day. Circadian rhythms affect insulin requirements over a 24-h period and these differences can be more pronounced in pubertal patients [36]. Pumps certainly allow for better accommodation of differences in circadian rhythm than is possible with a long acting insulin administered once to twice daily. Insulin pumps allow for precise delivery of small insulin doses with some pumps even allowing for dosing changes down to increments of 0.01 units and minimal insulin delivery of 0.025 units per hour which may be helpful in very young patients who are often very sensitive to insulin. Furthermore, some providers have argued that use of U10 (1/10th the concentration) insulin instead of standard U100 insulin in insulin pumps could improve precision by allowing delivery of very small doses [37]. However, others argue that insulin delivery is not truly continuous, but is delivered frequently in small boluses to mimic endogenous basal production. Therefore, the option for diluted insulin or very small basal dose increments may not change care in clinical practice. 

When considering insulin pump therapy, accuracy of insulin delivery is also an important consideration. Insulin pump manufacturers specify delivery accuracy to ±5% of insulin dose entered by using an international standard method. Typically these evaluations look at an average of the accuracy over 100 consecutive boluses within the system of interest. A 2013 study looked at insulin delivery accuracy of three conventional insulin pumps and one patch insulin pump by testing bolus accuracy in these systems. They noted a statistically significant difference in accuracy between the traditional systems and patch insulin pump with better single-dose accuracy noted in the traditional insulin pumps [38]. A standard measure of insulin pump accuracy, IEC 60601-2-24, helps to provide consistent testing of systems, but some researchers argue that this measure may be incomplete and alternative measures are being proposed [39].

Most importantly, pumps have been shown to improve glycemic control resulting in a lower HbA1c. In a recent study of pediatric patients with T1DM, patients with insulin pump therapy were shown to have lower rates of severe hypoglycemia, lower incidence of diabetic ketoacidosis, lower HbA1c, and lower total daily insulin doses than matched patients on multiple daily insulin injection therapy [40]. Insulin pumps allow patients increased flexibility in their routines and account of insulin action time thereby reducing the risk of insulin stacking. Temporary basal rates can be used to decrease hourly insulin during or after intense exercise. Patients are able to give multiple doses without requiring additional injections and utilize variable bolus settings for high fat meals. In the following sections, currently available insulin pumps are introduced and Table 2 provides a comparison of systems available within the US.

### 4.1. Medtronic Insulin Pumps

Medtronic was founded initially in 1949 as a medical device repair shop. In 1983, they released their first insulin pump to the market, the Minimed 502. Over the next several years, they launched updates to insertion sites and infusion sets that allowed for temporary detachment from the pump system. In 2003, they introduced the BolusWizard which wirelessly shared the glucose value from the system-integrated glucometer and suggested an insulin dose. In 2006, they released the MiniMed Paradigm Real-Time System that integrated CGM data for viewing on the insulin pump display. In 2013, the FDA approved the MiniMed 530G with Enlite for patients greater than age 16 years. This was the first system available in the US that provided ‘Threshold Suspend’ automation and would stop insulin delivery for up to two hours if the glucose level reached a preset low limit with no user response to the alert. The 630G was released in 2016 and was followed quickly by their most recent insulin pump the Medtronic 670G System [41].

The Medtronic 670G system features two operational modes: manual mode in which the system acts as a traditional pump, and auto mode in which the system acts as a hybrid closed loop device using CGM input to alter basal insulin output. The Medtronic 670G system will be discussed in greater detail below.

### 4.2. OmniPod Insulin Pumps

Insulet Corporation, the maker of OmniPod, was founded in 2000 and first received FDA clearance for OmniPod in 2005. This system was the first developed ‘patch’ insulin pump with two components: a disposable OmniPod infusion pump and the OmniPod Personal Diabetes Manager (PDM) [42]. The PDM is a handheld battery-powered device that controls insulin delivery from the disposable infusion pump. The infusion pump can be worn for up to 3 days and is waterproof up to 25 feet for 60 min. It will deliver insulin based on the programmed basal rates set up in the PDM. The PDM communicates wirelessly with the infusion pump and needs to be within five feet to give a bolus insulin delivery or change previously set basal insulin delivery rates.

The most recent iteration, OmniPod DASH, is anticipated for wide availability in early 2019 [43]. This system uses Bluetooth technology for communication between the PDM and infusion pump and allows users to view their PDM data on mobile phone applications. Additionally, with the smartphone applications, users will be able to share data with up to 12 people. The application will integrate Dexcom G6 CGM data with insulin delivery data on a single screen for streamlined analysis of information. However, the user will still need the PDM to make basal rate delivery changes or to give bolus insulin doses.

### 4.3. Tandem Insulin Pumps

In 2008, Tandem Diabetes Care, Inc. was founded from a previous company Phluid, Inc. [44]. The t:slim insulin pump received FDA clearance in 2011 and was the first ever touchscreen insulin pump. In 2015, they received FDA approval for integration with the Dexcom G4 CGM system. The t:slim, via Bluetooth technology, offered Dexcom G4 CGM data display on the insulin pump screen. This allowed for data to be interpreted in real-time with only one device. The newest Tandem product, the X2, was released in 2016. 

The Tandem X2 insulin pump features integration with Dexcom G5 or G6 CGM systems. They use Basal-IQ technology to reduce incidence of hypoglycemia in patients using both the X2 insulin pump and a Dexcom G6 CGM. The PROLOG trial showed significantly reduced rates of hypoglycemia without rebound hyperglycemia with use of the Tandem Basal-IQ system. The algorithm works by predicting glucose levels 30 min into the future and suspends insulin delivery if the predicted glucose value is <80 mg/dL or the actual glucose value is <70 mg/dL [45]. Updates of the Tandem insulin pump will be through software updates that can be downloaded to the patient’s pump as new systems are released.

### 4.4. Insulin Pumps Systems Available Only Outside of the US

#### 4.4.1. Cellnovo Insulin Pumps

The Cellnovo system is an insulin pump available to patients in Europe, but not currently available in the US. The system is comprised of a small insulin pump that is controlled via Bluetooth technology with a locked down Android device. The pump has short tubing and uses an adhesive to attach to the skin. It is water resistant to a depth of 36 feet for up to 1 h. This system also includes an activity tracker and food library, as well as applications for data management [46].

#### 4.4.2. Kaleido Insulin Pumps

The Kaleido insulin pump is newly available in Europe at this point. Some of this pump’s unique features include the option of placing the infusion site with variable lengths of tubing to accommodate for different locations on the body and the insulin pump can be placed on the skin with an adhesive or placed in a pocket. Also, the system comes with two rechargeable pumps that can be used interchangeably and a handset that controls insulin delivery via Bluetooth technology. The pumps are water resistant up to a depth of 3.3 feet for up to 1 h; however, the pump charging station and handset must be kept dry. Kaleido also offers 10 color options for the insulin pump [47]. 

#### 4.4.3. Roche Diagnostics Accu-Chek Insulin Pumps

The Accu-Chek Combo system first became available in the US in 2012 following approval by the FDA. The system was comprised of an insulin pump and glucometer that was connected via Bluetooth technology. The Accu-Chek insulin pumps are no longer supported in the US; however, they are still prominent in Europe and continue to support glucometers within the US. Their most recent systems, the Accu-Chek Spirit Combo and Accu-Chek Insight, are currently supported in many countries around the world and offer some advantages. The systems, comprised of an insulin pump and smart glucometer, allow the patient to change pump settings and use a bolus calculator without directly changing settings on the pump by input through the Bluetooth connected glucometer. The Accu-Chek Insight also allows patients to use pre-filled insulin cartridges instead of filling cartridges manually when replacing insulin [48].

#### 4.4.4. Sooil Dana Diabecare Insulin Pumps

The Sooil Company has two insulin pumps currently on the market, Dana Diabecare R and Dana Diabecare RS insulin pumps. They are currently available in multiple countries in Europe and Asia. The Dana Diabecare R model features a connected glucometer and lightweight design. The system also allows for remote control of pump settings through an Android based smartphone application [49]. The Dana Diabecare RS system was released in 2018 and connects with smartphone applications for both Android and iOS systems via Bluetooth technology [50].

### 4.5. Limitations

Insulin pumps have revolutionized insulin delivery by allowing for very precise insulin dosing and small dose delivery, but there are limitations to therapy. Cost is an important issue in care of patients with T1DM. In the DIAMOND study, researchers enrolled 75 adults with T1DM already using CGM and randomized them to either insulin pump therapy or continued multiple daily injections (MDI) therapy and followed these patients for 28 weeks [51]. They reported total per person costs for the length of the trial as $8272 for patients in the CGM + insulin pump group and $5623 for patients in the CGM + MDI group. Insulin pump therapy is often covered by insurance with improvement of out of pocket costs, but has associated monthly costs for supplies required for pump therapy including infusion sets, tubing, adhesives, batteries, insulin reservoirs or cartridges, and insulin. 

At this point, many insulin pumps are considered ‘open-loop’, meaning that the device acts based on settings that have been programmed into the system and cannot alter its output based on the effect of insulin on glucose. This requires careful monitoring of glucose values during therapy and a well-trained patient or parent who can alter insulin pump settings to vary insulin delivery based on clinical need. 

With insulin pump therapy, there has always been a concern for increased risk of diabetic ketoacidosis (DKA), a life-threatening complication of T1DM. Since insulin pumps utilize short-acting insulin, with pump site failure or disconnection, patients would not have the safety of subcutaneously injected long acting insulin to prevent DKA. In 2007, a consensus statement from the European Society for Paediatric Endocrinology issued a warning that ‘individuals using continuous subcutaneous insulin infusion (CSII) are potentially at increased risk of developing DKA, with DKA rates varying from 2.7–9 episodes per 100 patient-years’ [52]. More recently, Dogan et al. shared data from 205 adult T1DM patients treated with insulin pump therapy from 2006 to 2015. They noted only 10 cases of DKA in nine of their patients throughout the course of the study which indicates an incidence of 1.0 case/100 patient years which is reassuring [53]. Additionally, Hoshina et al. noted similar risk of DKA between patients using insulin pump versus multiple daily injection methods. Interestingly, they reported significantly lower rates of DKA in insulin pump patients who received care at a practice with experience caring for more than 250 insulin pump patients [54].

### 4.6. Future Directions

Over the last 50 years, insulin pumps have continued to advance and improve now with more precision and convenience than ever before. As these systems continue to evolve, more closed-loop systems will emerge onto the market. Multiple closed-loop systems are currently in development with unique algorithms designed to deliver insulin based on glucose levels measured with a connected CGM system. 

Bihormonal therapy with glucagon and insulin working together has long been discussed as ideal to mimic the endogenous functioning pancreas. The iLet Bionic Pancreas which plans to utilize both glucagon and insulin has been in development for several years [55]. In a randomized controlled trial, bihormonal pump therapy was compared with insulin only pump therapy showing improvement in mean blood glucose and reduction in incidence of hypoglycemia [56].

## 5. Hybrid Closed-Loop Insulin Delivery Systems

With the emergence of the first commercially available hybrid closed-loop system, the Medtronic 670G, diabetes care advanced a step closer to development of an artificial pancreas. These systems have been shown to increase the percentage of time in glucose range for users. In a 2010 study, OmniPod and Freestyle Navigator CGM were used to compare an open- versus closed-loop system showing an improvement from 64% to 78% time within range for users [57]. Additionally, multiple studies have shown reduction in incidence and severity of hypoglycemia in patients using closed-loop systems in both pediatric and adult patients [58,59]. Multiple algorithms are under development for use in hybrid closed-loop systems. Release of new hybrid closed-loop systems including the OmniPod Horizon, the Tandem Control-IQ, and the Beta Bionics iLet, is anticipated in the next couple of years.

There are currently four main types of control algorithms being employed in the development of closed loop systems. Model predictive control (MPC) algorithms are the most commonly used and aim to predict glucose levels in the near future and adjust insulin delivery based on that prediction. Another algorithm is the proportional integral derivative type which responds to real-time measured glucose values. Fuzzy logic algorithms determine insulin doses based on CGM data in the way that an expert clinician would make insulin adjustments. There are also bio-inspired algorithms that utilize a mathematical model to determine insulin doses based on how β cells would act in response to glucose in the body [60].

### 5.1. Currently Available

#### Medtronic

The Medtronic 670G is the only hybrid closed-loop system currently available on the market and is approved in the US and Europe in patients 7 years and up. This hybrid system uses the Guardian 3 sensor as well as SmartGuard technology during Auto mode to adjust insulin basal rates to help keep glucose levels within target range. Basal insulin delivery is adjusted every five minutes based on CGM readings to increase time within range. Additionally, the suspend before low feature, that can also be used in manual mode, stops insulin up to 30 min prior to reaching a preset low glucose value. The insulin delivery will automatically restart when glucose levels have recovered to avoid rebound hyperglycemia.

The system requires patients to enter carbohydrate corrections at meal-times similar to a traditional pump when in Auto Mode. For CGM operation and use in Auto Mode, patients must calibrate with finger-stick glucose values every 12 h and when alerted by the pump to do so. However, at this time, the system does not allow for sharing of real time CGM data with friends or family. In their pivotal trial, they reported a decrease in average Hb A1c values from 7.4% to 6.9% and an increase in time within desired glucose range (70 mg/dL to 180 mg/dL) from 66.7% to 72.2% of the time [61]. Stone et al. provide a further review of the Medtronic 670G Hybrid Closed-Loop System [62].

### 5.2. Anticipated in the Next 1–2 Years

#### 5.2.1. Beta Bionics

Beta Bionics is currently working on the iLet system that would allow for infusion of both insulin and glucagon in response to CGM data. The company is working with Senseonics Eversense CGM which is a physician subcutaneously implanted device that would read glucose levels for up to 3 months [29]. Initial release of the system is anticipated in 2020 as an insulin-only device with addition of glucagon anticipated in the next couple of years. In their 2017 study, they noted glycemic differences in the bihormonal treatment group versus traditional pump therapy including average CGM glucose of 140 mg/dL vs. 162 mg/dL and only 0.6% vs. 1.9% of time spent with glucose less than 60 mg/dL. They did, however, note an increase in complaints of nausea based on their scale scoring system [56].

#### 5.2.2. OmniPod

The OmniPod Horizon Automated Glucose Control System is under development. The system uses Dexcom G6 CGM input and Insulet personal MPC algorithm. They presented the results of their clinic trial at the 2018 American Diabetes Association meeting, showing increased time in range and decreased hypoglycemia during both day and night in adults, adolescents, and children. Their initial study showed mean time less than 70 mg/dL 0.7% in adults and 2% in pediatric patients and percentage of time in the target range 69.5–73% in adult and pediatric age groups [63]. The system release is anticipated in 2019.

#### 5.2.3. Tandem

In 2016, Tandem announced the plan for development of a closed-loop insulin delivery system called Control-IQ using Tandem X2 insulin pumps, Dexcom G6 CGM system, and TypeZero artificial pancreas (AP) technology [64]. Their initial pilot study published in December 2018 included five adult patients with well-controlled T1DM and showed mean time in range of 86% overall, mean glucose of 130 mg/dL, and median time less than 70 mg/dL of 2.8% [65]. They have recently completed another clinical trial of the Control-IQ system and will likely submit results for FDA approval in the coming months. The new system is anticipated later in 2019.

### 5.3. Limitations

The current technology allows for closed-loop insulin delivery response to glucose values and trends. However, at this point, users still need to check glucose values at least twice daily to calibrate the CGM device used in conjunction with the closed-loop system. Additionally, currently available systems require users to manually give bolus insulin for meal times by entering in carbohydrates and the current glucose value from either CGM or finger-stick value. 

For the currently available system the Medtronic 670G, options for glucose goals are limited while in Auto Mode to 120 mg/dL or 150 mg/dL. For many pediatric patients who experience hypoglycemia following exercise, an elevated glucose target of 150 mg/dL may be insufficient to prevent hypoglycemia. Conversely, for older patients who can achieve very tight glycemic control, a target of 120 mg/dL may be higher than desired. Patients have adapted to the system by giving ‘fake’ carbohydrate boluses to achieve tighter control; however, this practice could lead to increased risk of hypoglycemia in the user [66].

### 5.4. Future Directions

Hybrid closed-loop systems are still new to the market with multiple new systems anticipated in the coming years. Across the globe, several systems are currently in development or undergoing clinical trials and may be several years away from introduction into the market. Previous reviews have detailed systems in development extensively including the papers from Trevitt et al. and Bekiari et al. and should be referenced for a more detailed overview of anticipated systems [60,67]. As this technology continues to develop further, systems will ideally require less and less management from the patient and the provider. As systems move forward, length of time they may be worn will likely increase. Additionally, integrated CGM systems will require less calibration and significantly less finger-sticks. 

As these systems continue to progress, meal-time insulin may no longer be required and therefore the patient would not be required to enter carbohydrates consumed. Studies have started to look at unannounced meals in the setting of hybrid closed-loop insulin delivery systems. In a study done by Cameron et al., patients wore a closed-loop system in a hospital setting and a hotel setting and challenged the system with unannounced meals and exercise. During the study, they were able to report daytime mean glucose of 158 mg/dL showing promising results for a future system [68]. Forlenza et al. further investigated with both adults and adolescents in a hotel setting. In their study, they tested an Android-based AP system with six adults and 4 adolescents in the setting of exercise and unannounced meals. Although they showed overall mean CGM data of 157 mg/dL throughout the study, they did note a significantly lower CGM value following announced versus unannounced meals at 140 mg/dL versus 197 mg/dL. Meal announcement is still superior in glycemic control at this time given current insulin pharmacokinetics, CGM accuracy and lag, and possible speed of insulin delivery in current systems [69].

In recent studies, bihormonal pump therapy has shown improvement in mean blood glucose and reduction in incidence of hypoglycemia in comparison with insulin only pump therapy [56]. In the pursuit of bihormonal pump development, glucagon still is unstable in extended time periods in liquid form. In the future, we will likely see the emergence of a bihormonal closed-loop system using both insulin and glucagon, possibly as a standard of care.

## 6. Conclusions

Type 1 Diabetes is a chronic disease requiring glucose monitoring and intensive insulin therapy. Diabetes technology has continually evolved over the years to improve quality of life and ease of care for affected patients. Continuous glucose monitors allow patients to observe and discern trends in their glycemic control. These devices also give peace of mind with preset alerts for hypoglycemia and the ability for family or friends to follow a patient’s glucose trends. The major products available and in use today are the Abbott Freestyle Libre, the Dexcom G6, the Medtronic Guardian Sensor 3, and the Senseonics Eversense.

Insulin pumps have continued to improve and innovate since their emergence into the market. Pumps allow patients to infuse insulin for up to 3 days without giving individual insulin injections. These devices allow for very low doses in pediatric patients and varied basal rates during different times of day and during different activities. They also allow for more freedom in dietary schedule and multiple carbohydrate-based doses in addition to mealtimes. Hybrid closed-loop systems have harnessed the data gathered with CGM use to aid in basal insulin dosing and hypoglycemia prevention. As technology continues to progress, patients will likely have to enter less and less information into their pump systems manually. 

In the future, we will likely see a system that requires no manual patient input and allows users to eat throughout the day without counting carbohydrates or entering in any blood sugars. As technology continues to advance, endocrinologists and diabetes providers need to stay current to better guide their patients in optimal use of emerging management tools.

## Figures and Tables

**Table 1:** An overview of currently available CGM systems.

	Abbott Freestyle Libre	Dexcom G6	Medtronic Guardian Sensor 3	Senseonics Eversense
FDA Approved Age	18 years and up	2 years and up	14 to 75 years	18 years and up
MARD	9.4% [20]	9% [21]	8.7% [22]	8.8% [23]
Duration of use	14 days	10 days	7 days	90 days
Calibration	not required	not required, but can if desired	2 required daily, recommend 3–4 daily	2 required daily
Display options	Scanner	Receiver, Smartphone, Tandem X2; May share to 5 devices	Medtronic insulin pump, Smartphone; May share to 5 devices	Smartphone; May share to 5 devices
Warm up time	1 h	2 h	2 h	24 h
Alarms	no	yes	yes	yes
Approved insertion sites/procedure	arm	abdomen (2 years and older); upper buttocks (2–17 years)	abdomen	implanted in office, upper arm

**Table 2:** An overview of currently available insulin pump systems within the US.

	Medtronic 670G	OmniPod DASH	Tandem X2
Ages approved	7 years and above	All ages	6 years and above
Dosing increments	Basal: 0.025 units/hour; Bolus: 0.025 units	Basal: 0.05 units/hour; Bolus: 0.5 units	Basal: 0.001 units/hour at greater than 0.1 units/hour; Bolus: 0.01 units at greater than 0.05 units
Tubing	Tubing lengths—18”, 23”, and 32” Can be disconnected from infusion site	Patch, Tubeless	Tubing lengths—23”, 32”, and 43” Can be disconnected from infusion site
CGM integration	Medtronic Guardian Sensor 3	Dexcom G5/G6	Dexcom G5/G6
Hypoglycemia prevention	Yes, low glucose suspend with Sensor 3	No	Yes, Basal-IQ with G6 only
Closed Loop Available	Yes, Auto-mode	No	No
